# Instruments Measuring Externalizing Mental Health Problems in Immigrant Ethnic Minority Youths: A Systematic Review of Measurement Properties

**DOI:** 10.1371/journal.pone.0063109

**Published:** 2013-05-21

**Authors:** Carmen H. Paalman, Caroline B. Terwee, Elise P. Jansma, Lucres M. C. Jansen

**Affiliations:** 1 Department of Child and Adolescent Psychiatry, VU University Medical Centre, Amsterdam, The Netherlands; 2 Department of Epidemiology and Biostatistics, EMGO+ Institute for Health and Care Research, VU University Medical Centre, Amsterdam, The Netherlands; 3 Medical Library, VU University Medical Centre, Amsterdam, The Netherlands; Tehran University of Medical Sciences, Iran (Republic of Islamic)

## Abstract

**Background:**

Little is known about reliability and validity of instruments measuring externalizing mental health problems in immigrant ethnic minority youths.

**Aims:**

To provide an overview of studies on measurement properties of instruments measuring these problems in immigrant ethnic minority youths, their methodological quality and results.

**Methods:**

A systematic review of the literature in MEDLINE, EMbase, PsycINFO and Cochrane Library was performed. Evaluation of methodological quality of studies found was done by using the ‘COSMIN-checklist’. Full text, original articles, published in English after 1990 were included. Articles had to concern the development or evaluation of the measurement properties of self-reported, parent-reported and/or teacher- or clinician-reported questionnaires assessing or screening externalizing mental health problems in immigrant ethnic minority youths. Specific results of analyses on (an) immigrant ethnic minority group had to be given.

**Results:**

Twenty-nine studies evaluating 18 instruments met our criteria. Most studies concerned instruments with known validity in Western populations, tested mainly in African Americans. Considering methodological quality, inequivalences between ethnicities were found, self-reports seemed to perform better, and administration of an instrument influenced reliability and validity.

**Conclusion:**

It seems that the majority of instruments for assessing externalizing problems in immigrant ethnic minority youths is currently not sufficiently validated. Further evaluating existing instruments is crucial to accurately assess and interpreted externalizing problems in immigrant ethnic minority youths.

## Introduction

Externalizing problems are relatively common in children and adolescents [1], [2]. Externalizing mental health problems, such as Attention Deficit Hyperactivity Disorder (ADHD), Oppositional Defiant Disorder (ODD) and Conduct Disorder (CD), are particular problematic because their characteristics (e.g. aggression, lying, high levels of hyperactivity) not only affect the individual, but also the family and the wider community. Furthermore, externalizing problems are associated with many poor outcomes later in life, such as impairments in academic and psychosocial functioning, delinquency and substance abuse [3]–[7]. Immigrant ethnic minority youths are believed to have an increased risk of developing mental health problems [8]–[13]. Indeed, in many Western countries, immigrant ethnic minorities display behaviors that may be attributed to externalising problems. We use the term immigrant ethnic minorities to refer to those with a history of migration and are part of an ethnic of racial minority group in the country that they live in. This also includes African Americans, although they are considered to be distinct racial group rather than an ethnic minority group. According the US Census Bureau, Black or African Americans are those having origins in any of the Black racial groups of Africa. African Americans have a long history in the US. Some African American families have been in the US for many generations; others are recent immigrants from places such as Africa, the Caribbean or the West Indies [14]. Studies usually do not make a distinction between those with a long history in the US and recent immigrants, they simply refer to Black or African Americans. We therefore include African Americans in our review. Although we acknowledge the differences between racial and ethnic minority groups, for better readability we use the term immigrant ethnic minority youth throughout this paper.

In many Western countries, immigrant or ethnic minority status is associated with an overrepresentation in crime, large school drop out and impaired psychosocial functioning [15]–[18]. At the same time, however, these youths are less often treated for mental health problems [19]–[21]. With an increasing influx of immigrants to the Western parts of the world, and the continuity of problems among those with a history of migration [22], [23], it is important to be able to offer these youths the help they need. In order to do so, an early and accurate assessment of externalizing problems is important.

However, most instruments assessing externalizing problems are based on Western (e.g. European, American, Australian) perspectives on child behavior and most validation data originate from European or Anglo-American culture [24], [25]. Ideally, the assessment of externalising problems should reflect the underlying construct, and should not be affected by group membership such as ethnicity or culture [26]. However, meanings of scores may not be identical for immigrant ethnic minority youths, as to those for whom the instrument has been developed [27]. According to Van de Vijver and Phalet [28], inequivalencies between scores may be associated with the level of acculturation, a process of cultural and psychological change that comes with immigration [8]. Such inequivalencies hamper the use and interpretation of instruments across different cultural groups. Although these inequivalencies are largely recognized in research, studies continue to rely on scores obtained without first testing the extent to which both the instrument and the meaning and structure of its underlying constructs are equivalent for the investigated group [29]. Despite the importance of accurate assessments in ethnic minority youths and theoretical assumptions of inequivalencies, an overview of research on validity and reliability of externalising problems assessments in ethnic minority youths is lacking. Therefore, this literature review aims to provide an overview of available published studies that *did* evaluate measurement properties of the assessment of externalizing problems in immigrant ethnic minority youths by means of questionnaires based on self- parent- teacher- and/or clinician reports. Providing such an overview may give directions for future research in terms of selecting an appropriate instrument based on available published studies, and the issues that should be taken into account when measuring externalizing problems in immigrant ethnic minority youths. In addition, an overview of available published studies evaluating these measurement properties may also provide information on the shortcomings in this area of research. With this review of literature, we hope to contribute to the knowledge and shortcomings of whether and how instruments should be adjusted for immigrant ethnic minority youths.

According to Van de Vijver en Poortinga [30], validity problems in cross-cultural research may occur at the levels of both content and construct. Good content validity means that all items from a questionnaire are relevant and form a complete and good reflection of the measured construct. Problems in content validity may occur because of cultural differences in societal structures, values and socialization practices. These differences may influence the meaning and/or structure of a measured construct and the perception of its related item content. Therefore, content validity requires a thorough knowledge of the society of origin [29].

Construct validity is the extent to which the scores of an assessment are truly a reflection of the construct to be measured. Problems with construct validity across immigrant ethnic minority groups may occur when there are different perceptions of meanings of an item. Such problems can be traced by (a) conducting factor analyses or using item response theory (structural validity), (b) investigating the degree to which the instrument correlates with other related or similar measures as expected within a system of theoretical relationships (concurrent validity or hypothesis testing) or (c) by testing the degree to which an instrument relates to some external criteria or a ‘gold standard’ (criterion validity or predictive validity). A specific aspect of validity in the assessment of problem behavior in youths is caused by the use of multiple informants. Since there is no ‘gold standard’ in mental health research, the use of multiple informants, such as teachers, is highly valued in screening and assessing psychopathology in youths [31]. This may be problematic in cross-cultural research, since there is evidence that teachers assign higher scores of externalizing problems to immigrant ethnic minority youths than to majority youths with similar problems [32]–[36].

Reliability problems in cross-cultural research are problems related to the method of testing. For instance, it has been found that there are ethnic differences in the use of ordinal rating scales, as well as yes/no categories. This is illustrated by findings that Hispanics and African Americans exhibit extreme checking on Likert-type scales [37], [38]. Reliability problems may of course also occur due to poor item translation or inappropriate content [30]. Information on reliability can be obtained by testing the interrelatedness among the items in a questionnaire (internal consistency, α) or conducting identical tests on the same population within a short time interval and/or with other raters (test-retest reliability, inter-rater reliability, intra-rater reliability).

It is clear that problems with validity and reliability affect the interpretation of test scores, prevalence rates, and developmental and intervention trajectories, highlighting the importance of information on the measurement properties of instruments in assessing and interpreting problem behaviors in immigrant ethnic minority youths. The aim of this study is therefore: 1. To provide an overview of published studies on measurement properties of questionnaires measuring externalizing mental health problems in immigrant ethnic minority youths. 2. To investigate the methodological quality of these studies. 3. To evaluate the results of these studies.

A systematic review was conducted, using the COnsensus-based Standards for the selection of health status Measurement INstruments (COSMIN) checklist. This checklist was developed in an international Delphi study as a multidisciplinary, international collaboration with all relevant expertise involved, in which international consensus was reached on terminology, definitions, and a taxonomy of the relationships of measurement. This checklist has recently become available and evaluates the methodological quality of studies on measurement properties of health status questionnaires [39] in a clear manner.

## Methods

This systematic review was conducted based on the ‘Protocol for systematic reviews of measurement properties’ from the Knowledge centre Measurement Instruments VUmc [40]. A systematic review of measurement properties is defined as a systematic review of all available studies on the measurement properties of all available measurement instruments that aim to measure a particular construct in a particular population (www.cosmin.nl). Quality assessment of the studies included was done by means of the COSMIN checklist. This checklist is specifically developed for the quality assessment of studies on measurement properties [39], [41].

### Search Strategy

We searched the following databases from 1990 to April 2012 (initial search to November 2010; update search to April 2012) to find studies on measurement properties of instruments, assessing externalizing mental health problems in immigrant ethnic minority youths: MEDLINE, EMbase, PsycINFO, Cochrane Library. We used four blocks of index terms to search the databases. The first block referred to *mental health* with terms like: “Mental Health”, “Mental Disorders”, “Psychopathology”, “Psychiatric”. Since we were not sure if there were enough studies to review instruments on externalizing problems separate from internalizing problems, we did not make a distinction between externalizing and internalizing problems at this stage. The second block referred to *children and adolescents* with terms like: “Child”, “Adolescent”, “Youths”, “Teens”. The third block referred to *ethnic minorities* with terms like: “Emigrants and Immigrants”, “Ethnic Groups”, “Minority”. The forth block consisted of a previously developed search filter for finding studies on *measurement properties* [42], including terms like: “Psychometrics”, “Validation Studies”, “Internal consistency”, “Discriminant analysis”, “Factor analysis”. An example of the search strategy is provided as a supplement file ( File S1). After it became clear that there were enough articles to narrow down and only focus on externalizing problems we decided to do so.

### Selection Criteria of Included Studies

A study was included if it was published in English after 1990. Only full text original articles were included. Abstracts, reviews and editorials were excluded. The study had to concern the development or evaluation of the measurement properties of questionnaires assessing or screening externalizing mental health problems in immigrant ethnic minority youths. That means that both diagnostic instruments and behavior scales assessing or screening externalizing problems were included. Instruments with subscales of externalizing problems were included. However, in general, the total scores of an instrument are in presented, since usually no specific information of the subscales was available for immigrant ethnic minority youths. Studies on refugees and asylum seekers were excluded, since these populations are usually characterized by a temporarily stay and often faced with specific difficulties regarding mental health problems, such as severe trauma and depression [43], [44]. Studies on self-reports, parent reports as well as teacher and clinical expert reports of externalizing behavior were included. Specific results of analyses on (an) immigrant ethnic minority group had to be given, although results presented from other populations were no reason for exclusion. Instruments on drug abuse and delinquency are framed within national legislations. Moreover, although drug abuse and delinquency may be symptoms of externalizing behavior, the symptoms itself are uninformative about externalizing problems. Therefore, studies on drug use were excluded as well as studies on delinquency, except if drug use or delinquency was part of an instrument measuring externalizing problems. Studies focusing only on the predictive value of a questionnaire, without studying specific measurement properties were excluded. Two reviewers independently assessed the titles and abstracts of the studies retrieved by the search (CP and LJ). In case of disagreement, there was discussion in order to reach consensus.

### Quality Assessment of the Studies

The COnsensus-based Standards for the selection of health status Measurement INstruments (COSMIN) checklist, an instrument to evaluate the methodological quality of studies on measurement properties of health status questionnaires, has recently become available [39]. We used the COSMIN checklist to determine the methodological quality of the studies included. The COSMIN checklist consists of nine boxes concerning methodological standards of reliability and validity for how each measurement property should be assessed [45]. Each box consists of several items (5–18), including items on design requirements and items on statistical analyses, which are scored on a four-point rating scale (i.e. “poor”, “fair”, “good”, or “excellent”). The COSMIN checklist includes guidelines for rating of each item [46]. In the articles included in this review, two reliability parameters (internal consistency and reliability) and five validity parameters (content, criterion and three construct validity measures: structural, concurrent and cross-cultural validity) were reported.

***Reliability*** was defined as the extent to which scores are the same for repeated measurement under different conditions: e.g. using different sets of items from the same questionnaire (internal consistency) or over time (test-retest). In this review reliability was scored for the following measurement properties:

Internal consistency: The interrelatedness among the items in a questionnaire, mostly expressed by Cronbach’s α [39], [41]. Quality of the assessement of internal consistency was scored by 11 items, e.g. ‘Was the percentage of missing items given?’, ‘Was the unidimensionality of the scale checked?’ and ‘Was an internal consistency statistic calculated for each (unidimensional) (sub)scale separately?’

Reliability*:* The proportion of the total variance in the measurements which is due to ‘true’ differences between respondents [39]. Its quality assessment was based on 14 items, for instance: ‘Were there at least two measurements available?’ and ‘Were the test conditions similar for both measurements?’

***Validity*** is the extent to which a questionnaire measures the construct it is supposed to measure and contains the following measurement properties:

Content validity*:* The degree to which the content of a questionnaire is an adequate reflection of the construct to be measured [39]. Quality assessment of content validity was based on five items such as: ‘Was there an assessment of whether all items refer to relevant aspects of the construct to be measured?’ and ‘Was there an assessment of whether all items are relevant for the study population?’

Criterion validity*:* The extent to which scores on an instrument are an adequate reflection of a gold standard [39], scored by seven items including: ‘Can the criterion used or employed be considered as a reasonable ‘gold standard?’ and ‘Were there any important flaws in the design or methods of the study?’ and for dichotomous scores: ‘Were sensitivity and specificity determined?’

Construct validity is divided into three aspects: 1. *Structural validity:* The degree to which the scores of an instrument are an adequate reflection of the dimensionality of the construct to be measured [39]. Its quality was assessed by seven items, e.g. ‘Was exploratory or confirmatory factor analysis performed?’ and ‘Was the sample size included in the analysis adequate?’ 2. *Hypothesis testing or concurrent validity:* The degree to which a particular measure relates to other measures in a way one would expect if it is validly measuring the supposed construct, i.e. in accordance with predefined hypotheses about the correlation or differences between the measures [39]. Its quality was tested by ten items such as: ‘Was the expected direction of correlations or mean difference included in the hypothesis?’ and ‘Was there an adequate description provided of the comparator instrument(s)?’3. *Cross-cultural validity:* First, the degree to which the performance of the items on a translated or culturally adapted instrument is an adequate reflection of the performance of the items of the original version of the instrument. Second, the degree to which performance of the items (dimensionality) is similar across ethnic groups. This is assessed by multiple group factor analyses or Differential Item Functioning [39]. Third, the degree to which there is normative equivalence (measurement equivalence) across ethnic groups: e.g. does the same score, by teachers or parents for instance, on an instrument have the same meaning across different ethnic groups? Quality assessment of cross-cultural validity was measured by 15 items, e.g. ‘Were items translated forward and backward?’ and ‘Was differential item function (DIF) between language or ethnic groups assessed?’

Assessment of the methodological quality was performed by two independent reviewers (CT and LM). In case of disagreement between the reviewers, there was discussion with a third reviewer (HdV) in order to reach consensus.

### Best Evidence Synthesis – levels of Evidence

To summarize all the evidence on the measurement properties of the different instruments we synthesized the different studies by combining their results, taking the number and methodological quality of the studies and the consistency of their results into account. Levels of evidence are similar to those proposed by the Cochrane Back Review Group (see table 1) [47], [48]. The results of the studies were rated as positive or negative, based on criteria proposed by Terwee et al. [49].

**Table 1:** Levels of evidence.

Level	Rating	Criteria
strong	+++ or −−−	Consistent findings in multiple studies of good methodological quality OR in one study of excellent methodological quality
moderate	++ or −−	Consistent findings in multiple studies of fair methodological quality OR in one study of good methodological quality
limited	+ or −	One study of fair methodological quality
conflicting	+/−	Conflicting findings
unknown	?	Only studies of poor methodological quality

## Results

**Figure 1:**
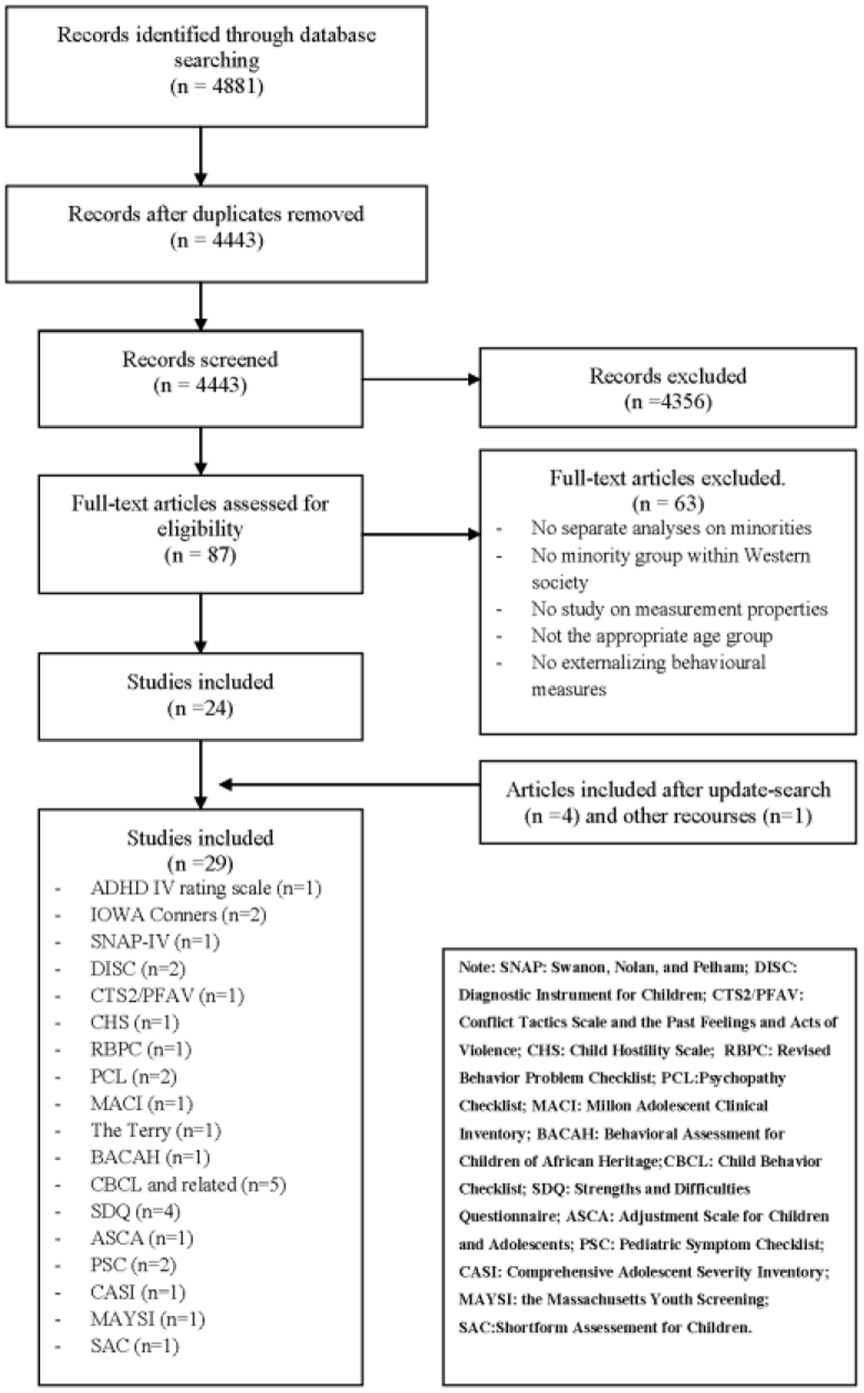
Flow Diagram search and inclusion.

As shown in figure 1, the search strategy resulted in a total of 4443 unique hits, from which 87 articles were selected based on titles and abstracts. Most excluded articles compared scores obtained from different countries without testing the questionnaire’s validity. The full text of these 87 articles was evaluated, resulting in 24 studies included in the 2010 search. In addition, four articles were included from the update search in 2012, resulting in a total of 28 articles that met our inclusion criteria. Additional reference search, resulted in one more included article, making a total of 29 included studies. The articles included evaluated 18 instruments concerning various constructs of externalizing mental health problems in ethnic minorities living in Western societies. Most articles were of US origin (n = 26), focusing on African American (n = 20) and/or Hispanic (n = 12) youths. Furthermore, three European studies were found: two Dutch studies, focusing on Moroccan, Surinamese and Turkish adolescents and one Norwegian study, focusing on Pakistani youths.

### Descriptives and Quality of the Studies

Table S1 shows descriptives of all studies included in our review. In addition, a summary of the quality of the measurement properties of the instruments is presented in table 2. The instruments included a wide range of externalizing concepts like ADHD, aggression, psychopathy and behavioral problems.

**Table 2:** Quality of measurement properties per questionnaire.

Instrument	Reliability				Validity			
	Internal consistency	Reliability	Content validity	Structural validity		Hypothesis testing/concurrent validity	Cross cultural validity	Criterion validity
DISC	na	++	na	− −		na	−	na
ADHD-IV rating scale	− − −	na	na	− − −		na	− − −	na
IOWA Conners	na	na	na	++		na	− − −	na
SNAP- IV	++	na	na	− −		na	− −	na
CTS2/PFAV	?	na	na	na		+	na	na
Child Hostility Scale	?	na	na	na		na	?	na
RBPC	na	na	na	na		na	−	na
PCL	?	+	na	+++		−	++	na
The Terry	?	+	na	na		na	na	na
BACAH	++	na	+++	++		na	na	na
MACI	?	na	na	na		+	na	na
CBCL	+	+	?	?		+/−	+/−	++
SDQ	+++	na	+++	+++		++	− − −	na
ASCA	?	na	++	?		+	?	na
PSC	na	?	na	na		?	?	?
CASI	?	+	na	?		?	?	?
MAYSI	++	na	na	++		na	+	na
SAC	++	+	na	++		++	?	?

+++ or − − − = strong evidence positive/negative result, ++ or − −  = moderate evidence positive/negative result, + or − = limited evidence positive/negative result, +/− = conflicting evidence, ? = unknown, due to poor methodological quality, na = no information available.

#### ADHD

Five studies that evaluated ADHD assessments were included in our review, evaluating four different instruments: The ADHD-IV rating scale, the IOWA Conners teacher rating scale (two studies), the Diagnostic Interview Schedule for Children (DISC; two studies, from which one specifically on the ADHD assessment of the DISC) and the Swanson, Nolan, and Pelham-IV (SNAP-IV). All five studies focused on African American youths.

ADHD-IV rating scale. In the study by Reid et al. [50], the school version of the ADHD-IV rating scale [51] was evaluated on internal consistency, structural validity and cross-cultural validity. This instrument consists of 18 items directly adapted from the ADHD symptom list as specified in DSM-IV [52]. There was strong evidence of internal inconsistency among African American and Caucasian youth, as well as strong evidence that the ADHD-IV rating scale lacks structural and cross cultural validity. Although differences were small, these results imply that teachers seem to have a different perception of ADHD in African American youth than in Caucasian youth as measured with the ADHD-IV rating scale.

IOWA Conners teacher rating scale**.** The IOWA Conners teacher rating [53] was evaluated in two studies. The IOWA Conners is a commonly used instrument for assessing ADHD and has two subscales: Inattention/Over activity and Aggression. In the study by Reid et al. [54], structural validity and cross-cultural validity were investigated. Although they found good validity, normative equivalence was questionable with higher scores for African American boys and girls as compared to European Americans. Comparable results were found in the study by Epstein et al. [55]. Focusing on cross-cultural validity, they found the same factor structures in both Caucasian and African Americans, but somewhat different hyperactivity factor loadings in African American females. Moreover, they found an additional factor of antisocial behavior in African American males. Differences in normative equivalence were also found, with teachers rating African Americans higher than Caucasians on externalizing scales. There is moderate evidence that the IOWA Conners has good structural validity on the main points, but again, there is strong evidence that this questionnaire lacks cross-cultural validity.

Diagnostic Interview Schedule for Children (DISC). The DISC is a widely used instrument, assessing DSM psychiatric symptoms and diagnoses in children, through parent interviews [56]. Two studies evaluated the DISC, of which one [57] specifically focused on ADHD by investigating structural validity and cross-cultural validity of the DISC on the ADHD scale. Results showed that perceptions of ADHD symptoms in African American parents differed from the norm group. There is moderate evidence that the DISC lacks structural validity and normative equivalence. Roberts et al. [58] investigated reliability of the full parent report of DISC2.1C in an African American and Hispanic sample. Methodological quality of the study was good and it revealed moderate evidence of similar reliability of the DISC2.1C across African American and Hispanic youths as compared to Anglo-American youths.

Swanson, Nolan, and Pelham-IV (SNAP-IV). The SNAP-IV was originally developed to assess ADHD symptoms according the DSM-III. Bussing et al [59] evaluated the short 26-item MTA version, named after its use in the Multimodal Treatment Study for ADHD [60], [61]. The questionnaire makes use of both teacher and parent ratings. Bussing et al [59] investigated internal consistency, structural validity and cross-cultural validity. Analyses revealed small differences in factor loadings between African American and Caucasian children of the teacher reports, while factor loadings for parent reports were equivalent across groups. Furthermore, they found a negligible effect of race in mean scores of parent ratings, and medium effect of race on teacher ratings, with higher ratings for African American children as compared to Caucasian children. Methodological quality of the study was good.

#### Aggression and conduct problems

Three studies focused on the assessment of aggression and conduct problems. All studies focused on Hispanic youths.

Conflict Tactics Scale (CTS2) and the Past Feelings and Acts of Violence. Although the study of Cervantes et al. [62] included two self-report violence risk assessments: the Conflict Tactics Scale and The Past Feelings and Acts of Violence [63], [64].We report them as one study, since only internal consistency and concurrent validity between the two measurements were investigated. They found both instruments to be reliable and valid in a sample of Mexican American high risk females. However, taking the methodological quality into account, limited evidence was found for concurrent validity.

Child Hostility Scale. Knight et al. [65] studied internal consistency and cross-cultural validity of the Child Hostility Scale [66]. In this study, the 28-item Child Hostility Scale was used to assess conduct problems in a sample of Hispanic and Caucasian children. Good internal consistency and cross-cultural validity was found for the Child Hostility Scale. However, methodological quality of the study was found to be poor.

Revised Behavior Problem Checklist (RBPC). Curtis & Schmidt [67] investigated cross-cultural validity of the Revised Behavior Problem Checklist (RBPC) by developing a Spanish translation of the instrument. The RBPC is a parent reported instrument to screen for conduct disorders, aggression and attention problems in children [68]. Some evidence was found for differences in vocabulary among different Hispanic subgroups.

#### Psychopathy

An often used instrument to assess psychopathy is the Psychopathy Checklist (PCL). This instrument is somewhat different from other included assessments, since it makes use of both clinical judgment, semi-structured interview and file review. Together it provides a standardized procedure and therefore we included the instrument in our review.

Psychopathy Checklist (PCL). The Psychopathy Checklist (PCL) was evaluated in two studies (one youth version and one revised version). Both studies focused on offending in youth. Before publication of the youth version [69], researchers had used modified versions of the PCL-Revised [70]. The study by Brandt et al. [71] on the revised version of the PCL revealed no differences in factor structures in Black American as compared to Caucasian youths. Methodological quality of the study was good for reliability and structural validity, but was poor for the assessment of internal consistency and concurrent validity. However, no differences on any points were found between the groups. The PCL-YV is a twenty item clinical rating tool to assess personal constructs of psychopathy among adolescents. Each item is rated by a clinician on the basis of a semi-structured interview and file review. Jones et al. [72] evaluated the PCL-Youth Version, and focused on the structural validity in a sample of African American and Hispanic youths as compared to Caucasian youths. The methodological quality of this study was excellent and outcomes revealed invariant factor structures across the three groups. Concluding, there is strong evidence that the PCL, both in the revised and the youth versions, has good structural validity across different ethnic groups. Furthermore, there is moderate evidence for good cross cultural validity of this instrument and limited evidence for its reliability across ethnic groups.

#### Behavioral problems

The majority of included instruments were general assessments of a broad range of behavioral problems. All included instruments were either focusing on externalizing problem behavior (e.g. Terry, Behavioral Assessment for Children of African Heritage (BACAH)), or included specific subscales of externalizing problem behavior (e.g. Child Behavior Checklist (CBCL), Strengths and Difficulties Questionnaire (SDQ), Millon Adolescent Clinical Inventory (MACI), Shortform Assessment for Children (SAC).

Terry**.** Bidaut-Russell et al. [73] investigated internal consistency and reliability of the Terry [74]. The Terry is a cartoon-like questionnaire which depicts African American children in various DSM-II-R-based situations, as well as socially approved behaviors. Bidaut-Russel et al. concluded that the Terry is a reliable and culturally sensitive instrument. Although methodological quality of the internal consistency assessment was poor, quality of the reliability assessment was found to be fair. Therefore, there is limited evidence that the Terry is a reliable instrument for assessing externalizing problems in African American children.

Behavioral Assessment for Children of African Heritage (BACAH). Lambert et al. [75] developed and studied internal consistency and structural and content validity of the Behavioral Assessment for Children of African Heritage (BACAH). This is an instrument on behavioral problems based on teacher, parent and self-reports, specifically developed for Black American youths. This study, of good to excellent methodological quality, gave moderate evidence for internal consistency and structural validity and gave strong evidence for content validity. Results indicate that the BACAH is a useful instrument in assessing behavioral problems in Black American youths.

Millon Adolescent Clinical Inventory (MACI). Blumentritt & VanVoorhis [76] evaluated the MACI [77], a widely used self-report assessment of adolescent psychopathology, in a sample of Mexican American boys. Taking methodological quality of this study into account, limited evidence was found for good concurrent validity of the MACI in Mexican American boys.

(Pictorial) Child Behavior Checklist and Behavior Problem Index (CBCL, PCBCL, BPI). The Child Behavior Checklist is a standardized parent report on children’s problem behavior [78]. The 118 problem items describe a wide array of problems, including externalizing problems such as aggression and rule-breaking behavior. Although this instrument has been evaluated in different countries [79]–[81], only four studies have evaluated the CBCL or related instruments such as the Behavior Problem Index (BPI) and the pictorial CBCL within ethnic minorities in a Western country.

Two studies evaluated the parent reported Child Behavioral Checklist for African American youths. Lambert et al. [82] evaluated the content validity of the CBCL by comparing records of clinical intakes with CBCL scores of African American youths. Findings suggest poor coverage of clinical problems by the CBCL in this group. Jastrowski Mano et al. [83] investigated internal consistency, and structural and concurrent validity and found a poor factor model fit and lower internal consistency in African American youths. However, methodological quality of the assessment of these parameters was poor. In addition, moderate evidence was found for lower correlations with other measures in African American youths compared to norm scores. However, a two factor model improved the model fit for this group.

Leiner et al. [84] evaluated the pictorial version of the CBCL as compared to the CBCL in a Hispanic sample. Internal consistency, reliability, concurrent and criterion validity were investigated. Findings support limited to moderate evidence that the pictorial version of the CBCL is a good alternative for the CBCL when there are communication barriers.

The BPI is modeled after the CBCL and was developed as a more convenient measure in length than the CBCL [85]. Two studies evaluated the BPI, both in Hispanic and African American youths, compared to Caucasian youths. Spencer et al. [86] investigated cross-cultural validity with fair methodological quality. The results of the study suggest that the BPI is not equivalent across ethnicity for all factor models. Items that were associated with this non-equivalence differed between Hispanic and African American youths.

In contrast, Guttmannova et al. [87], also focusing on cross-cultural validity in Hispanic and Black American youths, found moderate evidence for cross-cultural validity of the BPI. Although a poor factor fit was found, a revised factor structure based on the CBCL revealed inequivalence across ethnicity and conceptual and construct equivalence across the groups. Concluding, there is limited evidence of good internal consistency and reliability of the CBCL and related instruments across Black American and Hispanic youths. However, conflicting results were found regarding concurrent and cross-cultural validity, while moderate evidence of criterion validity was found. Several items had different loadings on the factors. However, with revised factor structures better fits can be established. The pictorial version of the CBCL can be used to replace the CBCL in Hispanic youths.

Strengths and Difficulties Questionnaire (SDQ). The SDQ [88] was investigated in four studies: two studies evaluated the self-report version and two evaluated the teacher-report version. The SDQ includes five scales of five items each, describing positive and negative attributes of children. Scales include externalizing problems such as conduct problems and hyperactivity. The four studies focused on various minorities: African American, Hispanic, Moroccan, Turkish, Surinamese, Pakistani and ‘other ethnicities’

Ruchkin et al. [89] focused on the structural validity of the self-report version in African and Hispanic urban youths compared to affluent suburban predominantly Caucasian youths. The study found strong evidence of equal factor structures across the groups. However, the study revealed good factor fit, but low factor loadings in all groups.

In line with these results, Richter et al. [90] found that the overall structure of the self-report version was the same in both Pakistani and ethnic Norwegians as well as in the group with ‘other’ ethnicities. However, thresholds and loadings differed for the minority groups. Therefore they recommend using the total scores instead of subscales. The methodological quality of this study focusing on cross-cultural validity was good.

The two studies on teacher report were both conducted by Zwirs et al. [91], [92] but different samples and methods were used in each study. However, both samples focus on Moroccan, Turkish and Surinamese children. The study from 2008 investigated content validity and is of excellent methodological quality. The 2011 study investigated internal consistency and cross-cultural validity and is of good methodological quality. Results from both studies revealed inequivalence across ethnicity as to content and structure. However, normative equivalence is questionable since means scores varied across ethnicity.

Concluding, there is strong support for good internal consistency, content, structural and concurrent validity of the SDQ self-report in Hispanic, African American, Pakistani and other ethnic minorities. However, strong evidence was found for normative in-equivalence in teacher reports across ethnicity.

Adjustment Scales for Children and Adolescents (ASCA). The ASCA contains 156 behavioral descriptions presented with reference to 29 specific social, play, or learning situations in which a child’s adjustment to authority and peers and various tasks may be observed [93]. McDermott [94] evaluated the ASCA in a sample of African American and, in addition, in a global sample of non-whites. This study presented the national standardization and validation of the ASCA. Moderate evidence was found for generalizability of the core syndromes in the African American sample and in the total group of non-whites, while limited evidence was found for concurrent validity in all studied groups. Concluding, although not strong, there is some support of ethnic generalizability of the ASCA.

Pediatric Symptom Checklist (PSC). Two studies evaluated the PSC [95]. The PSC is a 35-item questionnaire designed to be completed in the pediatrician’s waiting room by parents of 6-to-12 year old children. Murphy et al. [96] found the PSC to be reliable and valid in a sample of African American youth. However, methodology of the study was found to be of poor quality according the COSMIN checklist. Jutte et al. [97] focused on concurrent, cross-cultural, and criterion validity in a sample of Mexican American youths. In this study the CBCL was unwarrantly used as a ‘gold standard’, making the methodological quality of the study poor. However, lower sensitivity of the PSC in Mexican American youths was found.

Concluding, due to the poor methodological quality of the studies it is unknown whether the PSC is a valid and reliable instrument to use in African American and Mexican American youths. However, when accepting the CBCL as a ‘gold standard’, strong evidence was found for a lower sensitivity of the PSC in Mexican American youth.

Comprehensive Adolescent Severity Inventory (CASI). Meyers et al. [98] investigated the Comprehensive Adolescent Severity Inventory (CASI), a self-report instrument assessing chemical dependency, psychosocial functioning, delinquency and risk behaviors [99]. Internal consistency, reliability, and almost all measures of validity were investigated in an African American sample and in a sample of various minorities, compared to a sample of Caucasian substance abusing adolescents. No ethnic differences were found, but the study was on most aspects of poor methodological quality. Limited evidence was found for reliability of the CASI across the ethnic groups.

The Massachusetts Youth Screening (MAYSI). The Massachusetts Youth Screening (MAYSI) is a self-report instrument, specifically designed to assess mental health symptoms among youth in the juvenile justice system, and includes constructs such as alcohol and drug use and angry and irritable moods [100]. The instrument was evaluated in a study by Cauffman & MacIntosh [101] in a large sample of African American, Hispanic, Asian and Caucasian juvenile offenders. Internal consistency and structural and cross-cultural validity were investigated. No ethnic differences were found and the results gave moderate evidence of good internal consistency and structural validity of the externalizing scales of this instrument across ethnicity and limited evidence of good cross-cultural validity.

Shortform Assessment for Children (SAC).The SAC is a 48-item standardized and validated measure used to assess the overall mental health of children, including externalizing problems with a teacher or parent as informant. Tayson and Glisson [102] examined the cross-ethnic measurement equivalence of the SAC using parent reports in a sample of African American and White children referred to a juvenile justice and child welfare system. Moderate evidence was found for internal consistency, structural- and concurrent validity of the SAC in African American youths. Limited evidence was found for reliability of the SAC in African American youths. Although the authors report good results regarding concurrent and cross-cultural validity, methodological quality of these aspects was found to be poor. In conclusion, there is some evidence, that the SAC may be a valid behavioral rating scale for African American youths in the child welfare and juvenile system.

## Discussion

The aim of this study was to provide an overview of published psychometric studies on instruments measuring externalizing mental health problems in immigrant ethnic minority youths, to investigate methodological quality of these studies and to evaluate the results of these studies.

Regarding our first aim, we included 18 different instruments in 29 studies that met our search criteria. These instruments measured a wide range of externalizing concepts like ADHD, aggression, psychopathy and behavioral problems. Although these concepts have been investigated in multiple studies, only the SDQ and the CBCL were investigated in more than two studies. Moreover, all the instruments reviewed were investigated in a limited number of ethnic groups in only a few countries. The majority of the studies were from the US and focused on African American youths. Surprisingly few studies were conducted in European countries, even though in Europe the population of ethnic minorities is growing and no studies from Australia were included. The few European studies found were published recently, indicating that European countries have just begun investigating differences in measurement properties of instruments measuring externalizing mental health problems in immigrant ethnic minority youths. Still, considering the large minority populations in both the US and Europe, as well as in Australia, remarkably little research on reliability and validity of these instruments has been conducted. Our review points out a lack of knowledge of reliable and valid instruments in assessing externalizing mental health problems in immigrant ethnic minority youths.

Our second aim was to investigate methodological quality of the studies on measurement properties in order to interpret the results of these studies. By using the COSMIN checklist, we provided a clear overview of the measurement properties investigated and the methodological quality of these studies. First, it is encouraging that we found many studies of at least overall fair methodological quality. Nevertheless, several flaws were found. For instance, internal consistency was examined in twelve instruments, but in only six studies was methodological quality satisfying. Reliability was almost never investigated. As for validity, only three instruments were evaluated on content validity with satisfying methodological quality. However, most studies examined at least one or more validity properties, mostly structural and cross cultural validity.

Almost all the studies concerned instruments with known or assumed validity in Western populations that were tested in other ethnic groups. Exceptions were the Pictorial version of the CBCL (PCBCL), the Terry and the BACAH. This is in line with Van de Vijver [103], according to whom three different types of instruments in cross cultural research can be distinguished: 1. Instruments with known reliability and validity in Western groups for which empirical research is needed to find out whether the performance of the instruments is similar in other ethnic groups. 2. Instruments that are ‘culture free’ and can be used in diverse ethnic groups. Although there is debate if an instrument can be free from cultural influences, some instruments may be more suited for cross-cultural research than others. Since the language barrier is eliminated with the PCBCL, this instrument can be used reliably in a wider range of ethnic groups. In the study on Hispanics, internal consistency, reliability, and concurrent and criterion validity were good. Therefore, the PCBCL may be more suited to cross-cultural research than the written CBCL. 3. Instruments that are culture specific and developed for a specific ethnic group. The Terry and the BACAH are both culture specific instruments, developed or adjusted for African American children. The type of instrument may be guiding in what properties should at least be investigated. For instance, assuming that a particular instrument has been thoroughly tested on content validity and internal consistency in a Western population, by testing cross-cultural validity by means of multiple group factor analyses, information about the performance of an instrument in a different ethnic group can be established. For culture specific instruments, establishing content validity should be the first priority.

Our third aim was to evaluate the results of the studies. Based on this review we can not make firm recommendations on what instrument to use: as mentioned before, we found too many instruments that were tested in too small a number of studies, while the quality was not always satisfactory. However, in the following we attempt to draw some general conclusions based on the published studies included that may be indicative of the issues regarding measuring externalizing problems in immigrant ethnic minority youths.

First, it seems that ethnicity *does* matter in assessing externalizing mental health problems in youths, since a number of the studies lacked cross cultural validity. These results indicate that scores may have a different meaning in minority groups than in the majority groups. As a consequence, norm scores and cut-off scores often used in assessments should be established for every subgroup separately. At the least, researchers and clinicians should be aware of the fact that scores may need a different interpretation in ethnic minorities.

Second, self-reported measures may be more valid than teacher and parent reports. For instance, in this review good results were found for internal consistency, content, and structural and concurrent validity of SDQ self-reports in several ethnic groups, while in teacher reports normative equivalence was found to be questionable. Other studies also revealed validity problems in teacher and/or parent reports. Studies of the parent reported DISC, the teacher reported ADHD-IV rating scale, the teacher reported IOWA Conners and the teacher/parent reported SNAP-IV, all came to the same conclusion that there is poor cross-cultural and/or structural validity of these instruments measuring ADHD in African American youths: Teachers rated African American children generally higher on ADHD as compared to Caucasian children, while parents of African American children gave other ratings than expected based on norm scores. Furthermore, studies of the parent version of the CBCL reported ambiguous results concerning validity across ethnic groups. In contrast, some good results were found concerning validity and reliability in self-reported instruments. Good internal consistency and good structural and cross-cultural validity were found for the MAYSI. Other instruments such as the ASCA, CASI, CTS2, PFAV and SAC showed promising results, but were evaluated in only one study on very limited measurement properties within limited immigrant ethnic minority populations. The differences in validity between the informants is an important finding, since many child and adolescent assessments rely on multiple informants rather than solely self-reports, especially in young children [31]. However, in line with previous studies [33]–[36], [104], our results indicate that teacher ratings may be influenced by race-related beliefs and behavioral expectations. As for parents, it has been found that non-Western parents report fewer externalizing disorders as compared to Western parents [105], [106]. As stated earlier, acculturation may influence assessment outcomes [28]. Acculturation is usually conceptualized by two dimensions: culture maintenance and adjustment [8]. According to Van de Vijver and Phalet, adjustment to a host culture (regardless of culture maintenance) means that this person can be considered to belong to the population for which the instrument has been developed [28]. Possibly, youths are more ‘adjusted’ than their parents, and are therefore more comparable with the population for which the instrument has been developed. The assessment of acculturation, they state, should therefore be part of assessment in immigrant ethnic minority groups.

Third, the way an instrument is administered may be a factor to take into consideration. First, semi-structured interviews conducted by professionals may have an advantage in overcoming ethnicity differences as compared to structured questionnaires, since semi-structured interviews give a trained professional the opportunity to probe in more detail. The PCL for instance, a semi-structured interview for measuring psychopathy in offenders, seems to perform well in several immigrant ethnic minority groups with good reliability, as well as structural and cross-cultural validity. Second, by using pictures instead of written instruments, language problems can be eliminated. Examples are the Terry and Pictorial CBCL. Third, the use of instruments specifically developed for immigrant ethnic minority groups may be useful. The Terry was specifically developed for African American children, as was the BACAH. Although there are indications that these instruments perform well, main problem with these kind of specifically developed instruments remains that they are not useful when comparing outcomes with other ethnic groups.

Several limitations in this study should be acknowledged. First, a relatively small number of studies was found that fulfilled the inclusion criteria. We performed a systematic review of published scientific studies that reported on measurement properties of instruments measuring externalising problems in immigrant ethnic minority youths. Although we thoroughly assessed all titles and abstracts of all retrieved articles, including articles that not mainly focused on measurement properties, only 29 studies met our inclusion criteria. However, the relatively small number of found studies is also a meaningful result: It reflects the current lack of studies on measurement properties of instruments measuring externalizing problems in immigrant ethnic minority youths.

Second, like in every systematic review, results presented may be biased due to the fact that research with ‘negative’ or ‘uninteresting’ results is less likely to be published [107]. Furthermore, only papers in English were included, creating a potential language bias. In addition, unpublished work and manuals were not included, and information may have been missed. However, such sources are hardly accessible or public. Even more important, these sources are not peer reviewed, making their result hard to interpret and of questionable meaning to implement in a systematic review.

Third, the studies included in the systematic review reported on different populations of immigrant ethnic minority youth with a very diverse background, making it impossible to add up the results of all included studies. Nevertheless, while focusing on measurement properties, we were able to discuss some of the overall findings.

Fourth, problems regarding the assessment of externalizing problems are just one aspect of many problems regarding immigrant ethnic minority youth and mental health care. For instance, the under-representation of immigrant ethnic minority youth in mental health care because of a higher treatment threshold, lower access rates and lower parental detection because of health literacy are important issues [19], [20]. It would be interesting to investigate associations between these issues and how the assessment of externalizing problems is conducted.

Although, based on the current review, it can not be concluded that the majority of assessments on externalizing mental health problems in immigrant ethnic minority youths are conducted with instruments that have not been sufficiently validated in this population, it can be concluded that currently there is little published scientific evidence that supports reliable and valid use of such instruments. None of the instruments included has been evaluated on all relevant measurement properties and only few immigrant ethnic minority groups were included in the evaluated studies. Consequently, this may seriously hamper the interpretation of results of assessments.

Investing in research on the measurement properties of such instruments and making results available for the scientific community would mean important progress in the research on externalizing mental health problems in minorities, producing more valid and reliable results in both assessments and research. Studies on related topics, such as cross-country research may provide valuable information for giving direction to further research. For instance, extensive work regarding cross-country validity has been conducted regarding Achenbach System of Empirically Based Assessment (ASEBA) [108] and the SDQ [109].

Researchers and clinicians need reliable and valid instruments to identify problems in order to target effective interventions. As long as information on the reliability and validity of such instruments is not available, investing in evaluating existing instruments on reliability and validity and publication of the results is crucial.

## Supporting Information

Table S1 **Characteristics of the included studies.**
(DOCX)

File S1 **Example search criteria PubMed.**
(DOCX)
