# Supplementary material for: Instruments Measuring Externalizing Mental Health Problems in Immigrant Ethnic Minority Youths: A Systematic Review of Measurement Properties
Source: PLoS One. 2013 May 21;8(5):e63109. doi: 10.1371/journal.pone.0063109 (PMC3660354; doi:10.1371/journal.pone.0063109)
Supplement: Table S1 — Characteristics of the included studies. (DOCX) [file pone.0063109.s001.docx]

**Supplement Table S1. Characteristics of the included studies**

| **Authors** | **Instrument** | **Ethnicity/**  **race** | **Sample characteristics** | **Measurement properties and methodological quality** | | **Summary findings** |
| --- | --- | --- | --- | --- | --- | --- |
|  |  |  |  |  | |  |
| Reid, DuPaul, Power, Anastopoulos, Rogers-Adkinson, Noll et al., 1998 | ADHD-IV Rating Scale: School Version, teacher rating | African- American | Male students (age 5-18), drawn from a normative sample of the ADHD-IV Rating Scale-School Version. African American (n=381) and Caucasian American (n=1359). | Internal consistency  Structural validity  Cross cultural validity | excellent  excellent  excellent | Similar, but no identical constructs of ADHD. No large single item difference, but total cumulative difference between African American and Caucasian male students. |
| Reid, Casat, Norton, Anastopoulos & Temple, 2001 | IOWA Conners: teacher rating | African- American | African Americans (n=2421) and European Americans (n=1874) aged 5 to 11 years, drawn from nine urban elementary schools. | Structural validity  Cross-cultural validity | good  good | Structural equivalence of IOWA Conners good. Normative equivalence questionable, with higher teacher rating of African American children as compared to European American children. |
| Epstein, March, Conners & Jackson, 1998 | IOWA Conners: teacher rating | African- American | African American (n=418) and Caucasians (n=609) from 10-16 years old from two junior high schools from a semi-rural county. | Cross-cultural validity | good | On main points same results for African Americans and Caucasians. However, African American males had an additional factor of antisocial behaviour. African American females had different hyperactivity factor loadings from Caucasian females  Teachers rated African Americans higher on externalizing scales. |
| Bussing, Fernandez, Harwood, Hou, Garvan & Eyberg | Swanson, Nolan, and Pelham-IV (SNAP-IV):  Parent and teacher rating | African- American | High risk sample of African American (n=88 and Caucasian (n=178) children derived from school registration records including African American (n=505) and Caucasian (n=1108) children. | Internal consistency  Structural validity  Cross-cultural validity | good  good  good | Small differences in factor loadings of teacher report. Small effect of race in mean scores of both parent and teacher ratings, with higher ratings for African American children. However, differences were found too small to stratify further analyses by race. |
| Hillemeier, Foster, Heinrichs & Heier, 2007 | DISC: parent report | African- American | Low SES, high risk children (age 8-9), primarily African American (n=542) and Caucasian (n=529). | Structural validity  Cross cultural validity | good  fair | Different parent perceptions of ADHD symptoms in African American parents as compared to Caucasian parents. |
| Roberts, Solovitz, Chen & Casat,1996 | DISC2.1C: parent report | African-American  Hispanic | Adolescents aged 12 to 17 years who were receiving psychiatric treatment in the Gulf Coast area of Texas. Time 1 sample size n=334; Time 1-Time 2 sample size n=299. | Reliability | good | Similar reliability across all groups. |
| Cervantes, Duenas, Valdez & Kaplan, 2006 | 1. The conflict tactics scale (CTS2)  2. Past Feelings and Acts of Violence scale (PFAV): self-reports | Mexican- American | Low risk (n=150) and high risk (n=150) female adolescents (14-18 years of age) from a predominantly low income Mexican American community. | Internal consistency Concurrent validity | poor  fair | Adequate reliability and validity of both instruments. |
| Knight, Virdin, Ocampo & Roosa, 1994 | Child Hostility Scale (among others) | Hispanic | English speaking Hispanic children (n=201) and Anglo- American children (n=536), mean age 10.3 years. | Internal consistency Cross-cultural validity. | poor  poor | Considerable cross-ethnic functional and scalar equivalence. |
| Curtis & Schmidt, 1993 | Revised Behavior Problem Checklist (RBPC): parent report | Hispanic subcultures | Parents from Hispanic subcultures (n=50) from the Children’s Home and Aid Society of Illinois. | Cross-cultural validity | fair | It is suggested that future researchers be sensitive to the differences in vocabulary among Hispanic subcultures. |
| Jones, Cauffman, Miller & Mulvey, 2006 | Psychopathy Checklist- Youth Version (PCL-YV): clinical rating based on semi-structured interview and file review | African-American  Hispanic | Caucasian (n=206), African American (n=443) and Hispanic (n=363) serious juvenile offending boys in two US cities with mean age of 15.9 years. | Structural validity  Cross-cultural validity | excellent  good | PCL-YV is invariant across race. Overall fit and equivalence of the factor structures across ethnicity. |
| Brandt, Kennedy, Patrick & Curtin, 1997 | Psychopathy Checklist (PCL-R): clinical rating based on semi-structured interview and file review | Black American | Sample of incarcerated mainly Black Americans (n=54) and Caucasian boys (n=22) between 13 and 19 years of age. | Internal consistency Reliability  Structural validity  Concurrent validity | poor  fair  good  poor | No race difference. Good structural and concurrent validity. |
| Bidaut-Russell, Valla, Thomas, Bergeron & Lawson, 1998 | The Terry, mental health cartoon-like screener for African American children: self-report | African-American | Convenience sample of inner city African American boys (n=36), mean age 8 years from Missouri and Texas. | Internal consistency Reliability | poor  fair | Good reliability. Culturally sensitive. |
| Lambert, Rowan, Kim, Rowan, An, Kirsch et al., 2005 | Behavioral Assessment for Children of African Heritage (BACAH): parent, teacher and self-report | Black American | Black American children (n=559) ages 4-18 on whom parents reported. Teachers reported on children (n=489) ages 5-18. Adolescents aged 11-18 reported on their own functioning (n=417). | Internal consistency Structural validity  Content validity | good  excellent  good | Results show that in its present form the instrument is useful for the assessment of Black American children. |
| Blumentritt & VanVoorhis, 2004 | The Millon Adolescent Clinical Inventory (MACI): adolescent interview | Mexican- American | Total group of Mexican American (n=131) adolescents at risk with a mean age of 15.5 years. | Internal consistency Concurrent validity | poor  fair | In general good internal consistency, with the exception of a somewhat lower than expected alpha of an impulsivity scale. |
| Lambert, Rowan, Lyubansky & Russ, 2002 | Child Behavior Check List (CBCL): parent report | African-American | Clinical records of African American youths (n=1605) from 4-18 years old from several clinics and hospitals. | Content validity | poor | For African American children the items of the CBCL may not provide adequate coverage of clinically relevant problem behaviours. |
| Jastrowski Mano, Hobart Davies, Klein-Tasman & Adesso, 2009 | Child Behavior Check List (CBCL): parent report | African-American | African American caregivers (n=145) who came to family intervention support and services. Parents reported on adolescents with a mean age of 14.5 years. | Internal validity  Structural validity Concurrent validity | poor  poor  good | Poor factor model fit; however, alternative two-factor model improved fit statistics. Lower internal consistency and lower correlations in African American adolescents with other measures than reported in normative sample. |
| Guttmannova, Szanyi, & Cali, 2008 | Behavior Problem Index (BPI), parent report. (modelled after the CBCL) | Hispanic  Black-American | Data from the National Longitudinal Survey of Youth. Hispanic (n=233), Black American (n=352) and White (n=514) children were followed biannually from 1990 (from 5 to 7 years of age) to 1994 (from to 9 to 11 years of age). | Cross-cultural validity | good | Poor fit, but equivalence across groups in revised factor structure based on CBCL. Conceptual and construct equivalence across groups. |
| Spencer, Fitch, Grogan-Kaylor & McBeath, 2005 | Behavior Problem Index (BPI), parent report (modelled after the CBCL) | Hispanic  African-American | Data from the National Longitudinal Survey of Youth. Hispanic (n=324), African American (n=477) and Caucasian (n=890) children between 4 and 14 years of age. | Cross-cultural validity | fair | Variance across the groups. Item level analyses identified the items associated with the inequivalence across ethnic groups. |
| Leiner, Rescorla, Medina, Blanc, Ortiz, 2010 Haack LM, Gerdes AC, Schneider BW, Hurtado GD: Haack LM, Gerdes AC, Schneider BW, Hurtado GD: | Pictorial version of the Child Behavior Checklist (PCBCL) | Hispanic | Mostly Mexican American (n=2335), low-income families with children between 6 and 16 years drawn from electronic records of patients attending clinics in El Pasa. | Internal consistency  Reliability  Concurrent validity  Criterion validity | fair  fair  good  good | PCBCL and CBCL show comparable test-retest, reliability and mean scores. Also comparable in discriminating problems. PCBCL is a good alternative when there are communication barriers. |
| Ruchkin, Jones, Vermeiren & Schwab-Stone, 2008 | Strengths and Difficulties Questionnaire (SDQ), self-report. | African-American  Hispanic | Urban sample of mainly African American (n= 2686) and Hispanic (n=1242) students, mean age 13 years, compared with affluent suburban students, predominantly Caucasians (n=785) with mean age 14 years. | Internal consistency Structural validity | excellent  excellent | Good fit but bad loadings, equal for both samples. |
| Zwirs, Burger, Schulpen & Buitelaar, 2008 | Strengths and Difficulties Questionnaire (SDQ), used as screening list, teacher report | Moroccan  Turkish  Surinamese | Dutch (n=69), Moroccan (n=77), Turkish (n=53) and Surinamese (n=55) children from inner-city low SES neighbourhoods with mean age 7.8 years. | Content validity | excellent | Based on four SDQ items in both Dutch and non-Dutch children, a good prediction of low or high probability of having externalizing disorders could be made. No ethnic differences. |
| Zwirs, Burger, Schulpen, Vermulst, HiraSing & Buitelaar, 2011 | Strengths and Difficulties Questionnaire (SDQ), teacher report | Moroccan  Turkish  Surinamese | Dutch (n=677), Moroccan (n=699), Turkish (n=437) and Surinamese (n=371) children from two large cities in the Netherlands. Data was taken from the Detection of ADHD among children from Different Ethnic Origins in the Netherlands Study (ADEON), carried out in 2002/2003. | Internal validity  Cross cultural validity | good  good | Invariant factor structure of the SDQ across ethnicity. Similar structures for Dutch and Surinamese teachers; however, mean scores on subscales varied across ethnicity. Normative equivalence questionable. |
| Richter, Sagatun, Heyerdahl, Oppedal, Roysamb, 2011 | Strengths and Difficulties Questionnaire (SDQ), self-report | Pakistani  ‘other’ | 15 to 16 year old 10^th^ graders. Ethnic Norwegians (n=5379), Pakistani (n= 516), ‘other’ (n=349). | Cross-cultural validity | good | Overall structure the same in the three samples, but thresholds and loadings differ for the minorities. Total scores preferable to subscales. |
| McDermott,  1993 | Adjustment Scales for Children and Adolescents (ASCA): teacher report | African- American,  ‘Non-whites’ | A norm sample plus supplementary validity samples aged 5-17 years. African Americans (n=251), Hispanics (n=266), Asians (n=22), other (n=29) and Caucasians (n=965). | Internal consistency Content validity  Structural validity  Concurrent validity  Cross-cultural validity | poor  good  poor  fair  poor | Support for generalizability of the core syndromes to African Americans and a global sample of non-whites. |
| Murphy, Reede, Jellinek & Bishop, 1992 | Pediatric Symptom Checklist (PSC): parent report | African-American | PSC from 6-12 year olds (n=123), selected from a clinic serving African American inner city children, compared with clinical interviews (n= 24). | Reliability  Concurrent validity  Criterion validity | poor  poor  poor | Good, valid and reliable. |
| Jutte, Burgos, Mendoza, Ford & Huffman, 2003 | Pediatric Symptom Checklist (PSC): parent report (self-report >11 yr) | Mexican-American | Children (n=210) between 4 and 16 years of age attending a health clinic for routine health care. | Concurrent validity  Cross-cultural validity Criterion validity | poor  poor  poor | Lower sensitivity in Mexican American children. A different cut-off score for clinical significance should be considered in Mexican Americans. |
| Meyers, Hagan, McDermott, Webb, Randall & Frantz, 2006 | Comprehensive Adolescent Severity Inventory (CASI): self-report | African-American varied ethnic minority groups | Substance abusing adolescents in treatment (n=205) aged 12-18 years. 60% White, 28% African American and 12% varied ethnic minority groups. | Internal consistency Reliability  Structural validity Concurrent validity  Cross-cultural validity Criterion validity | poor  fair  poor  poor  poor  poor | No differences between subgroups in generalizability of factor structure and internal consistency. |
| Cauffman & MacIntosh, 2006 | Massachusetts Youth Screening (MAYSI): self-report | African- American  Hispanic  Asian | Asian (n=243), African American (n=1101), Hispanic (n=1963) and White (n=599) juvenile offenders from 13-17 years of age. | Internal consistency  Structural validity  Cross-cultural validity | good  good  fair | For the externalising subscales no ethnic differences were found. |
| Tyson & Glisson, 2005 | Shortform Assessment for Children (SAC) | African-American | African American (n=562) and White youths (692), mean age 14.5, 65% boys referred to child welfare or juvenile system. | Internal consistency  Reliability  Structural validity  Concurrent validity  Cross-cultural validity | good  poor  good  good  poor | No differences between the groups. |
